# Development and verification of prognostic nomogram for ampullary carcinoma based on the SEER database

**DOI:** 10.3389/fonc.2023.1197626

**Published:** 2023-05-29

**Authors:** Nan Tang, Zeng-Yin Chen, Zhen Yang, He-Zhen Shang, Guang-Jun Shi

**Affiliations:** ^1^ Department of Hepatobiliary, Qingdao Chengyang District People’s Hospital, Qingdao, Shandong, China; ^2^ Dalian Medical University, Dalian, Liaoning, China; ^3^ Department of Hepatobiliary and Pancreatic Surgery, Qingdao Hospital, University of Health and Rehabilitation Sciences (Qingdao Municipal Hospital), Qingdao, Shandong, China; ^4^ Department of Hepatopancreatobiliary Surgery, Qingdao Municipal Hospital, Qingdao University, Qingdao, Shandong, China

**Keywords:** ampullary carcinoma, SEER, overall survival, nomogram, prognosis

## Abstract

**Background:**

Ampullary carcinoma (AC) is a rare cancer of the digestive system that occurs in the ampulla at the junction of the bile duct and pancreatic duct. However, there is a lack of predictive models for overall survival (OS) and disease -specific survival (DSS) in AC. This study aimed to develop a prognostic nomogram for patients with AC using data from the Surveillance, Epidemiology, and End Results Program (SEER) database.

**Methods:**

Data from 891 patients between 2004 and 2019 were downloaded and extracted from the SEER database. They were randomly divided into the development group (70%) and the verification group (30%), and then univariate and multivariate Cox proportional hazards regression, respectively, was used to explore the possible risk factors of AC. The factors significantly related to OS and DSS were used to establish the nomogram, which was assessed *via* the concordance index (C-index), and calibration curve. An internal validation was conducted to test the accuracy and effectiveness of the nomogram. Kaplan–Meier calculation was used to predict the further OS and DSS status of these patients.

**Results:**

On multivariate Cox proportional hazards regression, the independent prognostic risk factors associated with OS were age, surgery, chemotherapy, regional node positive (RNP),extension range and distant metastasis with a moderate C-index of 0.731 (95% confidence interval (CI): 0.719-0.744) and 0.766 (95% CI: 0.747-0.785) in the development and verification groups, respectively. While, marital status, surgery, chemotherapy, regional node positive (RNP),extension range and distant metastasis were significantly linked to AC patients’ DSS, which have a better C-index of 0.756 (95% confidence interval (CI): 0.741-0.770) and 0.781 (95% CI: 0.757-0.805) in the development and verification groups. Both the survival calibration curves of 3- and 5-year OS and DSS brought out a high consistency.

**Conclusion:**

Our study yielded a satisfactory nomogram showing the survival of AC patients, which may help clinicians to assess the situation of AC patients and implement further treatment.

## 1 Introduction

Ampullary carcinoma (AC) is the second most common periampullary malignancy (1). Over the past few decades, its incidence has increased due to the growing use of endoscopy and other inspection methods, as well as screening high-risk patients with adenomatous polyposis (FAP) (2, 3).

Since AC has different pathological origins and widely varying prognosis, it is essential to select the most beneficial treatment for the patient, which has a better chance of extending their survival. Although the TNM staging system, proposed by the American Joint Committee on Cancer (AJCC), is widely used in clinical practice to evaluate the prognosis of patients with AC, it only considers the depth of tumor invasion, the presence of distant metastases, and the number of positive LNs (4). It does not account for other crucial factors, such as age and tumor differentiation, which may impact a patient’s prognosis (5). Moreover, it predicts outcomes for populations, rather than individuals. Therefore, it would be valuable to develop a model that more accurately assesses the prognosis of AC patients.

Nomograms, also known as columnar graphing, were invented by Philbert Maurice d’Ocagne in 1884 to solve complex functions through graphical computation. They are widely used as predictive models in oncology and medicine. The ability of nomograms to generate individual probabilities of outcome events by integrating different prognostic-related factors satisfies our need for integrated biological and clinical models and enables the pursuit of personalized medicine.

Newer iterations of statistical software have made the calculation of nomograms easier and more convenient. However, the analysis of nomograms based on data from the patient population inevitably has inaccuracies in different populations. Increasing the sample size can be taken to minimize bias.

To the best of our knowledge, no nomogram analysis of overall survival and disease-specific survival in patients with AC has been conducted. Therefore, the purpose of this study is to develop and validate a novel nomogram that can accurately predict the survival of patients with AC.

## 2 Materials and methods

### 2.1 Patient datasets and study design

After registering an account and signing a data agreement on the SEER database website, we were authorized to download the data of AC patients using the SEER ∗ Stat version 8.4.0.1 software. We collected all available data on patients’ age at diagnosis, race, sex, marital status at diagnosis, surgery, surgery of lymph nodes, radiation, chemotherapy, regional nodes examined (RNE), regional nodes positive (RNP), lymph node ratio (LNR - defined as the number of RNP divided by the RNE), tumor size, extension range, AJCC stage, cause-specific death classification, and survival in months and vital status.

Patients’ prognosis was mainly evaluated based on the outcome of overall survival (OS) and disease-specific survival (DSS). We utilized the “caret” package of the R version 4.2.2 software to randomize patients into the development group (70%) and the verification group (30%).

The inclusion and exclusion criteria were consistent between the development and validation groups and are described below.

Inclusion criteria: (a) Primary site of the tumor: C24.1-Ampulla of Vater; (b) Year of diagnosis: 2004-2019; (c) Behavior code ICD-O-3: Malignant.

Exclusion criteria: (a) Unreported race recode; (b) Unreported AJCC stage; (c) Unreported tumor size; (d) Unreported grade; (e) Survival time mismatch with the year of diagnosis; (f) Patients with follow-up less than 1 month; and (g) Other variables that are unknown or missing from the database.

Notably, in our study, we focused on the eight types of pathology in AC patients, namely adenocarcinoma, villous adenocarcinoma, adenocarcinoma, intestinal type, adenocarcinoma with mixed subtypes, adenocarcinoma in adenomatous polyp, mucinous adenocarcinoma, signet ring cell carcinoma and other types that were combined and named as “others” based on the International Classification of Diseases for Oncology, Third Revision (ICD-O-3). The histological subtypes that were defined as “others,” in descending order of the number of patients, were adenocarcinoma in papillary carcinoma (8050), carcinoma (8010), cholangiocarcinoma (8160), adenosquamous carcinoma (8560), medullary carcinoma (8510), tubular adenocarcinoma (8211), somatostatinoma (8156), intraductal micropapillary carcinoma (8507), clear cell tumor (8005).

The term “localized” extension is used when the tumor is confined to the ampulla of Vater or when it extends into the sphincter of Oddi. The term “adjacent organs or tissues” is used when the tumor has invaded the following organs or tissues: hepatic artery and portal vein (primarily), gallbladder, colonic hepatic flexure, lesser omentum, liver (including the hilum), transverse colon of the stomach, and soft tissues surrounding the pancreas.

Because the patient population spans from 2004 to 2019, the AJCC staging in this study is the sixth edition. But we inferred the T-stage of the seventh edition AJCC based on the ‘Extension range’ and listed it as a new variable.

According to the AJCC staging of patients, all stage IV patients have distant metastasis, so stage IV patients are classified as Yes in the distance metastasis group

According to whether the patient has undergone lymph node dissection or not, the surgical methods of the patient are divided into radical resection and resection

Patients who do not identify as black or white are classified as “other,” while patients who are divorced, separated, widowed, or unmarried but living with a domestic partner are categorized as “ Single”.

### 2.2 Statistical analysis

Summary statistics for the study population are expressed as percentages or median values. The Mann-Whitney U was used as a test to analyze continuous variables with nonparametric distributions at the baseline. To plot nomograms, these continuous variables were transformed into categorical variables, and the optimal cut-off points for continuous variables were identified using the X-tile software (Rimm Laboratory, Yale School of Medicine, New Haven, CT, USA) for results-based optimization. These categorical variables were grouped based on clinical outcomes, and the chi-square test was used to determine correlations between them.

Independent risk factors were screened by univariate analysis (log rank) and forward stepwise Cox multivariate regression analysis using the “survival” and “plyr” packages in R version 4.2.2 software (The R Project for Statistical Computing, Vienna, Austria). and P values <0.05 were considered statistically significant.

Based on the independent risk factors obtained from cox regression analysis, the Nomogram was constructed using the ‘rms’ package in R software. To assess its accuracy, the Harrell’s C-index (concordance statistic or C-statistic) was calculated and the calibration curves for 3- and 5-year OS and DSS were plotted. A higher C-index indicates that the model prediction is more accurate. We performed internal validation using a random resampling procedure (bootstrapping) with 1000 resamplings to ensure the accuracy of the 3-year and 5-year calibrations when comparing predicted and observed OS and DSS. We also used Kaplan-Meier analysis to show the probable OS and DSS of the patients. Additionally, we compared the OS and DSS derived from the developed Nomogram and AJCC staging system using the ‘survminer’, ‘survival’, and ‘dplyr’ packages in R and evaluated them using the C-index.

We used the ‘nomogramEx’ package in R to calculate the score for each variable. Based on the nomogram score, we categorized patients into Low-A, Low-B, Medium-A, Medium-B, and High-A, High-B risk groups, and plotted Kaplan-Meier survival curves.

## 3 Results

### 3.1 Clinicopathological characteristics of the patient

According to our screening criteria, a total of 2409 patients participated in our study between 2004 and 2019. Of these patients, 1518 were excluded due to incomplete clinical information. Ultimately, 891 patients were enrolled in the follow-up study and randomized to either the development group (627 patients) or the validation group (264 patients).

In the development group, the male to female ratio was 1.31:1 (356/271). The median age of patients was 66. 9 years, and adenocarcinoma accounted for 74.0% of all carcinomas. The most common area of tumor invasion was pancreas, accounting for 30.0% of all cases. Most patients had tumors in the early stages of AJCC (I+II, 66.3%) with good differentiation (well + moderately differentiated, 63.0%). However, 284 patients died due to this disease, accounting for approximately 45.3% of the total number of patients. A summary of these patients’ characteristics can be found in **Table 1**.

**Table 1:** Characteristics of 891 patients suffered ampullary carcinoma in SEER^1^ database.

Factors	Development group	Validation group	p value
** Age, years [Median (SD)]**	66.9 (11.9)	67.4 (11.8)	0.670
**Sex**			0.685
Female	271 (43.2%)	118 (44.7%)	
Male	356 (56.8%)	146 (55.3%)	
**Race**			0.262
White	489 (78.0%)	197 (74.6%)	
Black	38 (6.1%)	17 (6.4%)	
Other^2^	100 (15.9%)	50 (18.9%)	
**Marital status**			0.748
Single^3^	228 (36.4%)	99 (37.5%)	
Married	399 (63.6%)	165 (62.5%)	
**Surgery**			0.182
None	65 (10.4%)	31 (11.7%)	
Radical resection	543 (86.6%)	219 (83.0%)	
Resection	19 (3.0%)	14 (5.3%)	
**LN Surgery^4^**			0.413
None	84 (13.4%)	45 (17.0%)	
1 to 3	39 (6.2%)	12 (4.5%)	
4 or more	504 (80.4%)	207 (78.4%)	
**Radiation**			0.221
No	470 (75.0%)	208 (78.8%)	
Yes	157 (25.0%)	56 (21.2%)	
**Chemotherapy**			0.347
No	318 (50.7%)	143 (54.2%)	
Yes	309 (49.3%)	121 (45.8%)	
**Grade**			0.478
Grade I	65 (10.4%)	21 (8.0%)	
Grade II	330 (52.6%)	141 (53.4%)	
Grade III	225 (35.9%)	101 (38.3%)	
Grade IV	7 (1.1%)	1 (0.4%)	
**RNE^5^[Median (SD)]**	12.3 (9.14)	12.3 (9.63)	0.904
**RNP^6^[Median (SD)]**	1.70 (2.69)	1.55 (3.01)	0.260
**LNR^7^[Median (SD)]**	0.136 (0.210)	0.115 (0.201)	
**Tumor size(mm)[Median (SD)]**	26.4 (41.6)	23.9 (14.0)	0.494
**Histologic Type**			**0.143**
Adenocarcinoma	464 (74.0%)	180 (68.2%)	
Adenocarcinoma in adenomatous polyp	16 (2.6%)	10 (3.8%)	
Infiltrating duct	11 (1.8%)	6 (2.3%)	
Adenocarcinoma, intestinal type	29 (4.6%)	12 (4.5%)	
Adenocarcinoma with mixed subtypes	5 (0.8%)	9 (3.4%)	
Mucinous adenocarcinoma	24 (3.8%)	17 (6.4%)	
Signet ring cell carcinoma	12 (1.9%)	7 (2.7%)	
Villous adenocarcinoma	44 (7.0%)	16 (6.1%)	
Others^8^	22 (3.5%)	7 (2.7%)	
**Extension**			0.028
localized	72 (11.5%)	50 (18.9%)	
Duodenal wall	184 (29.3%)	77 (29.2%)	
Pancreas	188 (30.0%)	66 (25.0%)	
CBD (Common bile duct)	47 (7.5%)	18 (6.8%)	
EBD (Extrahepatic bile duct)	6 (1.0%)	4 (1.5%)	
PST (Peripancreatic soft tissue)	**66 (10.5%)**	**28 (10.6%)**	
Adjacent organs or tissues^9^	64 (10.2%)	21 (8.0%)	
**AJCC stage**			0.273
IA	55 (8.8%)	39 (14.8%)	
IB	101 (16.1%)	39 (14.8%)	
IIA	77 (12.3%)	22 (8.3%)	
IIB	183 (29.2%)	81 (30.7%)	
III	170 (27.1%)	67 (25.4%)	
IV	41 (6.5%)	16 (6.1%)	
**T stage (AJCC 7th)**			0.033
T1	72 (11.5%)	50 (18.9%)	
T2	184 (29.3%)	77 (29.2%)	
T3	188 (30.0%)	66 (25.0%)	
T4	183 (29.2%)	71 (26.9%)	
**Distant metastasis**			0.790
No	586 (93.5%)	248 (93.9%)	
Yes	41 (6.5%)	16 (6.1%)	
**Cause specific death**			**0.495**
Alive or dead of other cause	343 (54.7%)	151 (57.2%)	
Dead (attributable to this cancer)	284 (45.3%)	113 (42.8%)	
**Survival months [Median (SD)]**	50.1 (44.5)	52.4 (50.2)	0.902
** Vital status**			0.514
Alive	202 (32.2%)	91 (34.5%)	
Dead	425 (67.8%)	173 (65.5%)	

^1^The Surveillance, Epidemiology, and End Results Program; ^2^includes patients whose race were not black or white; ^3^includes patients who are divorced, separated, windowed, and unmarried but have domestic partner; ^4^ Number of lymph nodes removed by surgery or aspiration; ^5^regional nodes examined; ^6^regional node positive; ^7^lymph node ratio (LNR - defined as the number of RNP divided by the RNE); ^8^based on the 3rd Edition (ICD-O-3), papillary carcinoma (8050), carcinoma (8010), cholangiocarcinoma (8160), adenosquamous carcinoma (8560), medullary carcinoma (8510), tubular adenocarcinoma (8211), somatostatinoma (8156), intraductal micropapillary carcinoma (8507), clear cell tumor (8005).^9^the tumor has invaded the following organs or tissues: hepatic artery and portal vein (primarily), gallbladder, colonic hepatic flexure, lesser omentum, liver (including the hilum), transverse colon of the stomach.

### 3.2 Overall survival and independent risk factors in the development group

The study had a median follow-up of 95 months (range 1-188). The median survival time was 37 months, with a 95% confidence interval of 47.0-53.6. The OS rates at 3 and 5 years were 50.5% and 40.5%, respectively. Univariate Cox analysis revealed that age, marital status at the time of diagnosis of the disease, surgery, LN surgery, grade, histologic type, RNE, RNP,LNR, tumor size, extension range, T stage, distant metastasis were all risk factors for OS. Although chemotherapy and radiotherapy were not statistically significant in univariate analysis, they are still the main treatment methods for advanced patients in clinical treatment, so both of them were also included in the multivariate analysis. The multivariate analysis showed that age, surgery, chemotherapy, RNP, extension range and distant metastasis were independent risk factors for OS (**Table 2**).

**Table 2:** Univariate and multivariate Cox analyses for OS based on ampullary carcinoma patients in development group.

Characteristics	Univariate analysis			Multivariate analysis			
	Score^1^	5-year survival (%)	p value	HR^2^	CI5	CI95	p value
Age							
20-53	53	53.7		1.000			
54-79	19	40.4	0.002	1.628	1.168	2.270	0.004
≥80	0	23.4	<0.001	2.671	1.799	3.966	<0.001
Surgery							
None	0	NA		1.000			
Resection	74	36.8	<0.001	0.321	0.168	0.614	<0.001
Radical resection	100	45.3	<0.001	0.2827	0.1672	0.4779	<0.001
Chemotherapy							
No	0	40.7		1.000			
Yes	39	38.5	0.275	0.631	0.484	0.821	<0.001
RNP							
0	70	51.5		1.000			
1 to 3	20	33.4	0.004	1.577	1.093	2.275	0.015
≥4	0	18.8	<0.001	2.380	1.395	4.061	0.001
Extension range							
localized	44	50.1	0.001	0.444	0.277	0.710	<0.001
Duodenal wall	43	54.2	<0.001	0.605	0.419	0.875	0.008
Pancreas	17	38.4	0.043	0.907	0.642	1.280	0.577
CBD (Common bile duct)	0	20.7	0.698	1.390	0.884	2.188	0.154
EBD (Extrahepatic bile duct)	38	33.3	0.341	0.296	0.102	0.856	0.025
PST (Peripancreatic soft tissue)>	10	23.3	0.829	1.117	0.738	1.690	0.601
Adjacent organs or tissues	8	18.9		1.000			
Distant metastasis							
No	31	42.3		1.000			
Yes	0	6.5	<0.001	2.287	1.512	3.460	<0.001

^1^Score calculated according to nomgram parameters; ^2^Hazard ratio.

### 3.3 Disease-specific survival and independent risk factors in the development group

The DSS rates for the development group at 1, 3, and 5 years were 82.1%, 61.1%, and 52.7%, respectively. Independent risk factors associated with DSS were marital status at the time of diagnosis of the disease, surgery, chemotherapy, RNP, extension range, distant metastasis (**Table 3**).

**Table 3:** Univariate and multivariate Cox analyses for DSS based on ampullary carcinoma patients in development group.

Characteristics	Univariate analysis			Multivariate analysis			
	Score^1^	5-year survival (%)	p value	HR^2^	CI5	CI95	p value
Marital status							
Married	19	58.7		1.000			
Single	0	42.0	<0.001	1.428	1.105	1.844	0.006
Surgery							
None	0	NA		1.000			
Resection	78	57.9	<0.001	0.165	0.063	0.434	<0.001
Radical resection	100	57.3	<0.001	0.164	0.082	0.327	<0.001
Chemotherapy							
No	0	55.8		1			
Yes	25	49.5	0.48	0.683	0.498	0.936	0.018
RNP							
0	65	64.5		1.000			
1 to 3	21	46.2	0.004	1.720	1.096	2.704	0.019
>4	0	25.8	<0.001	2.627	1.370	5.038	0.004
Extension							
localized	49	69.1	0.895	1.149	0.675	1.957	0.609
Duodenal wall	33	65.8	<0.001	0.517	0.333	0.802	0.003
Pancreas	9	47.5	0.866	0.461	0.156	1.366	0.162
CBD (Common bile duct)	1	29.4	<0.001	0.246	0.135	0.449	<0.001
EBD (Extrahepatic bile duct)	20	33.3	0.042	0.865	0.581	1.287	0.474
PST (Peripancreatic soft tissue)	9	38.0	0.439	0.911	0.561	1.480	0.705
Adjacent organs or tissues^6^	0	29.0		1.000			
Distant metastasis							
No	25	55.3		1.000			
Yes	0	8.1	<0.001	2.361	1.494	3.731	<0.001

^1^Score calculated according to nomgram parameters; ^2^Hazard ratio.

### 3.4 Prognostic nomogram for OS and DSS

**Figure 1:**
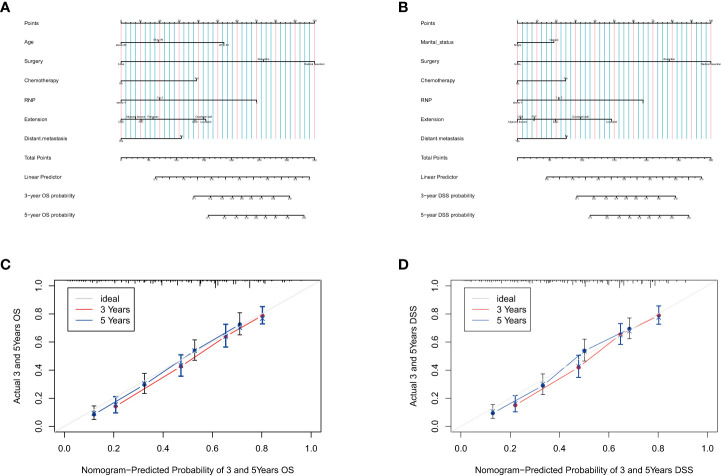
Prognostic nomograms for predicting the OS **(A)** and DSS **(B)** probability, and 3-and 5-year calibration curves of the development group when predicting the OS **(C)** and DSS **(D)**.

Based on the data from the development group, all independent risk factors associated with patients’ OS or DSS were enrolled in the prognostic nomogram, as shown in **Figures 1A, B**. Each factor was assigned a corresponding score, and the sum of scores reflected the OS, DSS, and mortality of patients in 3 and 5 years. The C-index of the nomogram for predicting OS based on the development group was 0. 731 (95% CI: 0.719-0.744), while the C-index for the validation group was Significantly higher at 0.766 (95% CI: 0.747-0.785). The predicted DSS nomogram showed better reliability and stability, with C-index values of 0.756 (95% CI: 0.741-0.770) for the development group and 0.781 (95% CI: 0.757-0.805) for the validation group. The C-index values of the two nomograms illustrated the reliability of the prediction models. The calibration curves for 3 and 5 years were consistent with the results of the C-index, indicating satisfactory consistency between the observation and prediction results for the OS and DSS of the development group (**Figures 1C, D**). Notably, the nomograms showed superior performance compared to the AJCC staging system (C-index of OS= 0.643, 95% CI 0.629-0.657; C-index of DSS=0.674, 95% CI 0.658-0.690; p<0.001).

### 3.5 Divide the risk level according to the nomograms’ score

**Tables 2**, **3** show the scores of each variable associated with OS and DSS. Using X-tile software, patients were divided into 6 separate groups based on the total score for their respective prediction purposes. According to the Nomogram score about OS, patients with a total probability score of <128, 128-175, 175-214, 214-260, 260-286, ≥286 were assigned to the High-A, High-B, Medium-A, Medium-B, Low- A, and Low- B groups, respectively. And for DSS, patients with scores <113, 113-157, 157-189, 189-214, 214-239, ≥239 were divided into six groups with the same name, respectively. **Figure 2** shows the Kaplan–Meier OS and DSS curves based on separated by nomogram-based groupings (A, C) and AJCC stage (B, D). The nomogram-based stage prediction was found to be better than that of the AJCC stage based on the predicted prognosis curves.

**Figure 2:**
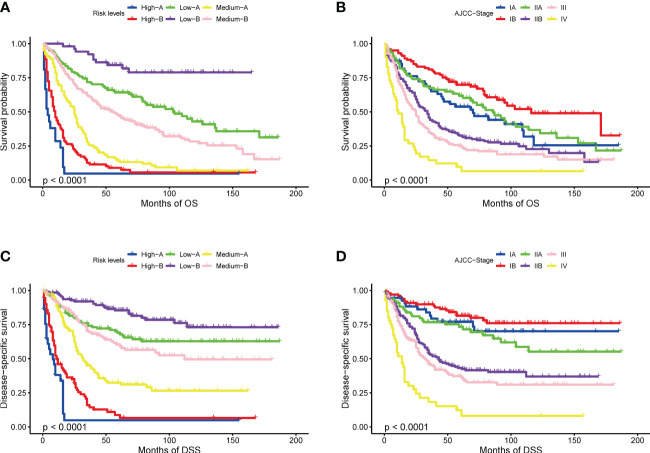
Kaplan-Meier curves of OS and DSS: based on risk levels **(A, C)**, based on AJCC stage **(B, D)**.

## 4 Discussion

The nomogram presented in this study was developed and validated using multivariate analysis, including age, surgery, chemotherapy, RNP, extension range and distant metastasis as independent risk factors for OS, and marital status at the time of diagnosis of the disease, surgery, chemotherapy, RNP, extension range, distant metastasis for DSS.

While patients may vary in hygiene, social status, and race, AC has been a rare gastrointestinal cancer, accounting for only 0.2% to 0.5% of cases in the past few decades (6, 7). AC is usually located at the end of the bile duct and often causes biliary obstruction at an early stage, leading to symptoms like jaundice. Consequently, it has a high surgical resection rate and better prognosis compared to other periampullary cancers (8). In spite of its rarity, clinicians have few reliable prognostic tools and limited understanding of AC prognosis.

Nomograms have become a popular tool in predicting patient prognosis for various types of cancers such as bladder, prostate, and penile cancers (9–11). By combining the nomogram with widely accepted cancer treatment guidelines, it can offer a more personalized assessment of a patient’s prognosis (12).

While nomograms are widely used to predict the prognosis of various cancers, the currently available nomograms for ampullary carcinoma (AC) mainly focus on postsurgical patients and those without lymph node metastases (13, 14). In this study, we focused on patients diagnosed with AC and analyzed their overall survival and disease-specific survival. Compared to existing nomograms, we found that age is closely related to the prognosis of AC patients. In particular, the median survival times of patients aged 20-54, 55-79, and above 80 were 65, 36, and 22 months, respectively, with a 5-year survival rates of 53.7%, 40.4%, and 23.4% (p<0.001). This finding may be attributed to the decline in organ function and resistance to stress, as well as an increase in comorbidities that come with aging.

Interestingly, we found that tumor size and pathological grade did not significantly affect AC survival rate in our multivariate analysis, possibly due to their collinearity. However, we did find that marital status was significantly associated with prognosis. Kaplan-Meier analysis revealed that married patients had a median survival of 45 months, with a 5-year survival rate of 43.7%, while single patients had a median survival of 26 months, with a 5-year survival rate of 33.5% (p<0.001), consistent with the findings of other studies. This result may be explained by various factors, including economic and environmental factors, as well as psychosocial factors (15). Having a partner or spouse often leads to a healthier lifestyle (16) and higher economic income, which may increase the chances of detecting the disease early and receiving active treatment. Moreover, the emotional support provided by a spouse can help reduce the negative effects of stress and improve treatment outcomes (17–20).

Regarding treatment modalities, both receiving surgery or not and the type of surgical modality were identified as independent risk factors for both OS and DSS. However, whether or not to receive chemotherapy or radiotherapy had little effect on patient DSS. This may be due to the fact that radical surgery is the recommended treatment for early-stage AC, whereas radiotherapy and chemotherapy are mostly used for patients with advanced AC or those who experience postoperative recurrence. These patients tend to have poorer survival status and survival time. Additionally, adjuvant chemotherapy and first-line chemotherapy programs are often tailored to the histological subtypes of the tumor. However, a retrospective study conducted by Ecker and colleagues found that the use of adjuvant chemotherapy did not improve clinical prognosis regardless of the type of pathology, be it intestinal or pancreaticobiliary (21). This suggests that most ACs are not responsive to chemotherapy and that treatment outcomes are not promising.

As the American Joint Committee on Cancer continuously updates its guidelines, the significance of RNP in determining disease prognosis is being recognized (4). Consistent with it, our study found that RNP was an independent risk factor for OS and DSS. In addition to this, it was emphasized that the number of surgically removed lymph nodes was also closely related to the prognosis. Complete clearance of regional lymph nodes helps to eliminate potential metastases, achieve effective R0 resection, reduce recurrence rate, and ultimately improve prognosis (22).

Our prognostic nomograms are based on the SEER database and includes a large sample size of AC patients from multiple disease centers, ensuring little relative bias and high confidence. There are the following advantages: Firstly, nomograms outperformed the AJCC staging system in predicting OS and DSS. This is reflected not only in its higher C-index value but also in the differential effect of different stages on prediction, as shown in **Figure 2**. This is probably because AJCC stage inclusion indicators lack individualization, such as age and marital status, which are not included in the stage criteria. Our prognosis models were more individualized and highlights the impact of surgical treatment and tumor invasion on the occurrence and development of the disease. Secondly, the variables used in the nomogram are easily obtained from the patient’s hospitalization information, and most of them can be obtained through preoperative imaging examinations. Therefore, they are also applicable to non-surgical patients. By using the variable score, clinicians can predict the prognosis accurately and evaluate the need for surgical treatment immediately. Thirdly, the clinical and pathological information of the nomogram is derived from the SEER database registered in 8 states in the United States, featuring multicenter clinical data. Therefore, the results should be more applicable to the general population than those derived from a single institution.

There are several limitations to the results of this study that need to be considered. First, as it was a retrospective study, there may have been confounding indications for chemotherapy and radiotherapy use, which could affect the results. The lack of effect of both on univariate analysis of OS and DSS may be due to selecting more patients with advanced and unfavorable disease. Second, the inclusion period of the study was quite long (2004-2019), which may have resulted in changes in surgical and pathological procedures over time, further affecting the prognostic significance of some parameters. Therefore, in the future, the disease-specific nomograms developed in this study should be externally validated using independent data sets to ensure their accuracy and reliability.

## 5 Conclusions

A disease-specific nomogram and overall survival nomogram were developed and validated for predicting the survival of patients with AC using commonly accessible clinicopathological characteristics. Both the development group and internal validation group had higher C-index values and better calibration curves. The nomogram scores allowed for clear grouping of patients according to their OS and DSS risk, with higher accuracy compared to AJCC staging. Patients in the high-risk group may require more aggressive postoperative treatment and closer follow-up, as they may have a poorer prognosis. Although this is a preliminary study, the nomogram shows promising results in predicting DSS and OS in patients with AC and should be further evaluated in future clinical studies.

## 6 Resource identification initiative

Surveillance epidemiology and end results (RRID : SCR_006902).

## Data availability statement

Publicly available datasets were analyzed in this study. This data can be found here: https://seer.cancer.gov/data/.

## Author contributions

NT: Data analysis and writing-original draft. H-ZS: Formal analysis. ZY: Validation. Z-YC: Writing - review & editing.G-JS: Methodology and supervision. All authors contributed to the article and approved the submitted version.
